# The Effectiveness of the Chronic Disease Self-Management Program in Improving Patients’ Self-Efficacy and Health-Related Behaviors: A Quasi-Experimental Study

**DOI:** 10.3390/healthcare12070778

**Published:** 2024-04-03

**Authors:** Ali Kerari, Ghareeb Bahari, Kholoud Alharbi, Latifah Alenazi

**Affiliations:** Nursing Administration and Education Department, College of Nursing, King Saud University, Riyadh 11421, Saudi Arabia; alikariri@ksu.edu.sa (A.K.); kalharbi1@ksu.edu.sa (K.A.); lalmodiani@ksu.edu.sa (L.A.)

**Keywords:** chronic disease, self-management, self-care, self-efficacy, health behaviors

## Abstract

The Stanford Chronic Disease Self-Management Program (CDSMP) is a valuable educational resource for supporting patients’ self-management behaviors. However, no evidence supporting its effectiveness in the Saudi Arabian population exists. Therefore, this study aimed to evaluate the effectiveness of the 6-month CDSMP in individuals with chronic conditions in Saudi Arabia within a primary care context. A quasi-experimental design was conducted in 110 adults living with ≥1 chronic disease in Saudi Arabia. The patients in the experimental group (n = 45) participated in a six-session CDSMP, whereas those in the control group (n = 65) continued their usual care. Baseline and 6-month assessments were conducted using relevant questionnaires to assess outcome measures. Analysis of covariance revealed that the participants who underwent the CDSMP had significantly higher self-efficacy levels in managing their conditions (F = 9.80, *p* < 0.01) and a greater tendency to adopt healthy behaviors to successfully manage their chronic illnesses (F = 11.17, *p* < 0.01). The participants who underwent the CDSMP also showed significant improvements in all health-related outcomes compared with those in the control group (*p* < 0.01). These findings indicated that the program had a positive effectiveness in self-efficacy, self-management behaviors, and health-related outcomes among adults with chronic diseases in Saudi Arabia. The CDSMP may be integrated into primary care settings to help patients successfully manage their chronic conditions.

## 1. Introduction

Chronic diseases pose a global challenge to human health, transcending geographical boundaries and socioeconomic status [1]. Chronic conditions such as diabetes, high blood pressure, and other ailments not only challenge individuals but also strain healthcare systems, increasing the financial burden on healthcare providers [2]. The global prevalence of these diseases increases owing to factors such as aging and unhealthy lifestyle choices, including poor dietary habits, lack of physical activity, and smoking [3]. If these diseases are left untreated, they can potentially overwhelm healthcare systems. Concerted efforts are necessary to curb the spread of these diseases or to self-manage them when they are already affected, thereby ensuring the health and resilience of societies [4].

A notable increase in chronic disease prevalence worldwide has been observed. Diabetes is highly prevalent worldwide, affecting a significant population proportion [5]. Similarly, high blood pressure, which is a major risk factor for cardiovascular disease, has a high incidence worldwide [6]. Respiratory conditions, particularly asthma, are widespread in areas with seasonal sandstorms [7]. Furthermore, the alarming prevalence of obesity globally is closely associated with numerous health conditions, particularly heart disease [8]. Mental health disorders and psychological ailments affect many individuals worldwide and pose a threat to their overall well-being and daily functioning [9]. These reports highlight the need for more effective preventive measures and appropriate management strategies for mitigating the impact of these diseases and fostering healthier and more resilient societies.

Saudi Arabia is no exception to the high chronic disease prevalence. Diabetes is the most common condition, with a prevalence of 23.7%, followed by high blood pressure [10,11]. Asthma affects individuals across all age groups, and Saudi Arabia regularly experiences sandstorms, significantly contributing to its incidence. With the implementation of Saudi Vision 2030, aiming to enhance various aspects of Saudi Arabia, continuous efforts are being made to improve the people’s health status. The healthcare sector also emphasizes appropriate health activities and programs to support preventive measures and effective management of chronic diseases [12]. In alignment with Vision 2030 goals, the Ministry of Health prioritized promoting healthy habits and increasing awareness as key objectives for fostering a healthy society.

When diagnosed with a chronic disease, embarking on a self-management journey is crucial to achieving a better and healthier life [13]. Adopting appropriate healthcare behaviors can help mitigate progression of the condition and decrease the risk of severe symptoms or mortality [14]. Ensuring the availability of reliable sources that promote healthy self-management behaviors is a global priority for healthcare systems to support patients in leading fulfilling lives. The Chronic Disease Self-Management Program (CDSMP) is a valuable educational resource [15]. The CDSMP is an evidence-based program specifically established to assist patients with chronic diseases in effectively managing their conditions, improving healthy behaviors, and reducing associated complications. The program offers vital information and skills related to self-management behaviors and fosters self-confidence in disease self-management [16]. Through various educational sessions employing techniques such as brainstorming and action planning, participants acquired valuable methods for enhancing self-management, adopting healthy behaviors, and developing practical problem-solving techniques.

The CDSMP plays a crucial role in equipping patients with the necessary tools and knowledge to successfully manage chronic diseases. The CDSMP sets itself apart from other programs owing to its evidence-based approach that supports a comprehensive focus on various aspects of patient care [15]. Compared with other programs, the CDSMP encourages patients to play a more proactive role in self-managing their health. Through its diverse educational methods and emphasis on informed decision-making, the program effectively enhances self-confidence and promotes better control over chronic diseases [17]. Moreover, the CDSMP is equipped with practical techniques to address disease symptoms and improve communication with healthcare providers, making the CDSMP a comprehensive healthcare program that surpasses programs that concentrate on specific behaviors [18].

The CDSMP can play an essential role in Saudi medical settings by addressing the specific needs of patients. Since the country experiences a high chronic disease prevalence influenced by cultural factors and lifestyle choices, such programs are invaluable to society. To date, there is no data on evaluating the efficacy of the CDSMP in the Saudi Arabian settings. Furthermore, some patients may have limited knowledge about essential self-management behaviors. Therefore, this study aimed to evaluate the effectiveness of the CDSMP in self-efficacy, self-management behaviors, and health-related outcomes in individuals with chronic conditions in Saudi Arabia. We hypothesized that the CDSMP may have a positive effectiveness in patients’ self-efficacy, self-management behaviors, and health-related outcomes.

This study utilized the principles of self-efficacy theory to explain and predict behavioral changes [19]. This theory encompasses performance accomplishment, vicarious experiences, verbal persuasion, and emotional arousal as guiding principles for behavioral changes. The CDSMP was developed based on the principles of self-efficacy theory, aiming to provide strategies for personal development, cognitive symptom management, problem-solving, and communication skills [18]. Given the applicability of these principles to patients with chronic conditions, this study employed them to enhance patients’ self-confidence and promote positive behavioral changes.

## 2. Materials and Methods

### 2.1. Study Design and Setting

A quasi-experimental, two-group, pretest–posttest was conducted. Two groups were formed; measurements were taken both before and after the intervention. Additionally, no participants were randomly assigned to either group [20]. This study was conducted in Riyadh, the capital of Saudi Arabia, which is experiencing rapid development across all sectors, including healthcare [21]. The city is renowned for providing the highest quality of care compared with hospitals in other regions. With an average population of approximately 7 million, Riyadh requires substantial medical capacity and top-notch healthcare services to meet its community health demands. Approximately 436 primary healthcare centers (PHCs) are dedicated to providing care for chronic and noncritical diseases [22].

### 2.2. Participants

The inclusion criteria were: (1) Saudi Arabians ≥ 18 years, (2) living with ≥1 common chronic condition(s) (diabetes, hypertension, asthma, heart disease, chronic obstructive pulmonary disease, or hyperlipidemia), and (3) receiving treatment at a PHC located in Riyadh. The exclusion criteria included active cancer treatment, active hepatitis C treatment, pregnancy, end-stage renal disease, dementia, and inability to participate. Patients with low literacy levels were excluded as they were likely unable to read or understand educational materials and survey forms.

A medium effect size is appropriate to examine the influence of behavioral interventions on chronic conditions [23]. Using the G*Power 3.1 electronic tool (Heinrich-Heine-Universität, Düsseldorf, Germany), a minimum sample size of 98 was required. To handle potentially missing data, which is common in longitudinal and experimental studies, an additional 10% of participants were included, leading to a new minimum sample size of 108.

### 2.3. Measures

Most instruments used in this study were guided by the Self-Management Resource Center (SMRC) to evaluate community-based interventions [24]. Demographic data (age, sex, marital status, education, income, and comorbidities) were collected at the baseline assessment. Three outcome domains were measured at the baseline and at 6 months, including self-efficacy, self-management behaviors, and health-related outcomes. The 6-month evaluation allowed for a more comprehensive understanding of the CDSMP’s lasting effects on the participants’ self-efficacy, self-management behaviors, and health outcomes. Changes in outcome measures could be used to evaluate the effectiveness of the program [25]. Qualitative interviews with some of the participants was another approach used to gather valuable insights into the participants’ experiences and determine the effectiveness of the CDSMP [26].

Self-efficacy scale: Self-efficacy was assessed using the Six-Item Chronic Disease Self-Efficacy Scale [27]. This tool included six items evaluating confidence levels in chronic disease management, including symptom management, role function, emotions, and communication with healthcare providers. Each item of this tool ranged between 1 and 10, with a higher score indicating a higher self-efficacy level [28]. The reliability was 0.91 in the original study and 0.92 in this study.

Self-management behavior scale: The Arabic-validated Self-Care Profile was used to assess self-management behaviors [11]. The Arabic-validated Self-Care Profile was a 4-point Likert scale with 19 items to assesses self-management behavior frequency on a scale ranging from (1 = rarely/never) to (4 = always), with higher scores indicating better self-management behaviors. The scale originally targeted common self-management behaviors, including medication compliance, physical exercise, healthy diet, smoking cessation, weight reduction, self-monitoring, regular doctor visits, stress reduction, and reduced alcohol consumption [29]. However, alcohol consumption was omitted from the validated Arabic version of the questionnaire due to its inappropriateness in the Saudi culture and alcohol consumption prohibition in the country [30]. Cronbach’s alpha was 0.84 in the validated version and 0.84 in this study.

Health-related outcomes scale: Health-related outcomes included cognitive symptom management, pain severity, illness intrusiveness, and depression. The Six-Item Cognitive Symptom Management Scale was used to assess the frequency of techniques applied by the participants to relieve pain, improve comfort, and reduce stress [27]. The six-items ranged between 0 (never) and 5 (always), with higher scores indicating higher cognitive symptom management levels. The reliability was 0.75 in the original study and 0.78 in this study. A five-item scale, a modified version of the Medical Outcomes Study, was used to assess pain over the past 4 weeks. Higher scores indicated more severe pain. In this study, Cronbach’s alpha was 0.84.

Illness intrusiveness was measured using an adapted version of the Illness Intrusiveness Rating Scale [31]. This scale comprised 13 items evaluating several domains, including health status, diet, work and finances, marital and family relationships, recreational and social relationships, and other life roles. The reliability was 0.86 in the original study [31] and 0.88 in this study. Depression was assessed using the Personal Health Questionnaire Depression Scale, in which the participants rated each item on a four-point scale from 0 (not at all) to 4 (nearly every day) [32]. The participants with a score of 10 were considered to have major depression, whereas a score ≥20 indicated severe major depression [33]. The reliability was 0.86 in the original study and 0.87 in this study.

### 2.4. Translation and Readability Assessment

All scales were translated into Arabic, the official language of Saudi Arabia, to facilitate participants’ understanding of the items. We engaged a fluent Arabic and English speaker with sufficient knowledge about the healthcare system in the country to perform the forward translation from English to Arabic. A backward translation was assigned to another individual with the same language proficiency and understanding of the Saudi healthcare system. Thereafter, the research team reviewed the translation steps to ensure their suitability for the Saudi culture. We then pilot-tested the questions in individuals with chronic conditions to assess their readability levels and gather suggestions for improvement. Finally, we confirmed that the participants adequately comprehended the items, indicating an acceptable readability level.

### 2.5. Procedures

The CDSMP was originally developed in English. However, we utilized the validated Arabic version to ensure its cultural and linguistic appropriateness for the Saudi community. It was administered for 2.5 h once a week over 6 weeks. Each workshop was facilitated by a professional and a lay leader (an individual with a chronic condition or the caregiver of a person with a chronic condition). The session topics included (1) techniques to cope with problems such as pain, fatigue, and stress; (2) physical exercise importance; (3) appropriate medication use; (4) communication techniques in healthcare settings; (5) the importance of healthy nutrition; and (6) new treatment plan evaluation [34]. The sessions were highly participative, and mutual support and success increased the participants’ willingness to manage their chronic conditions and maintain active and fulfilling lives.

The participants in the control group continued to receive their routine treatment at PHCs. These centers provided preventive and therapeutic services for noncritical cases and chronic diseases wherein simple medications were sufficient for treatment. In cases of critical condition or those requiring surgical intervention, the patients were transferred to hospitals for appropriate medical care.

The research team members were trained to facilitate the CDSMP workshops. Once they became eligible to use the program in Saudi Arabia, they recruited participants. This involved advertising on social media platforms and direct communication with officials from various primary care centers to obtain a list of individuals interested and eligible to participate in the study. Potential participants were informed about the study’s purpose, data collection procedure, confidentiality level offered, and their right to decline participation or withdraw from the study at any time. All patients who met the inclusion criteria provided informed consent.

The participants were then allocated to either the intervention or control group based on their natural grouping or preexisting conditions. The questionnaires were administered to the two groups at different time points. The first assessment was conducted before the first session began, and the second assessment 6 months after the completion of the 6-week program.

### 2.6. Intervention Fidelity

To ensure adherence to the CDSMP protocol, the program leaders received structured training from certified master trainers at the Stanford Self-Management Resources Center. Due to the required sample size in this study, 8 and 20 were the minimum and maximum acceptable number of participants in any workshop, respectively. The principal investigator and other research team members successfully managed all program-related aspects and data collection processes. To evaluate the program, the participants were asked to answer the following questions: (1) Have you applied any skills you learned from the CDSMP? (2) Have the skills you learned from the CDSMP helped you manage your medical condition? (3) Would you recommend the CDSMP for other patients with chronic conditions? Participants’ answers were given on a five-point Likert scale from 0 (not at all) to 4 (greatly). The responses were generally positive, indicating the effective utilization of the CDSMP.

To date, a few studies have adopted the Stanford model in regions such as China, Singapore, Germany, and Spain. These studies provided important recommendations for structuring cross-border adaptations and adopting a Stanford self-management intervention [35,36,37,38]. The CDSMP developed by Stanford University was adapted and implemented through a systematic process. The first step was to obtain a license to use the original CDSMP materials and reference books (English versions). Second, the principal investigator received a validated Arabic version of the Stanford Model from the SMRC. Third, the manual’s sections were evaluated via internal and external reviews for their use and appropriateness. The internal review included the research team, while the external review included healthcare providers and patients. Fourth, training facilitated by the SMRC was held for leaders from the research team in preparation for the program launch. After the leaders completed the training, the research team members were ready to conduct several workshops.

### 2.7. Data Analysis

SPSS (version 29) (IBM Corp., Armonk, NY, USA) was used for statistical analysis. Descriptive statistics are presented as numbers and percentages. When the normal distribution assumptions were met, data were analyzed using parametric tests. The mean values are used to present continuous variables. At the baseline, chi-square and independent *t*-tests were used to compare changes in demographic characteristics and outcome measures of the participants in both groups. A one-way analysis of covariance (ANCOVA) was performed to compare the changes in outcomes between the groups at 6 months, using the baseline score of the outcome measures as covariates. Statistical significance was set at *p* < 0.05.

### 2.8. Ethical Approval

This study was conducted in accordance with the Declaration of Helsinki, and approved by the Institutional Review Board of King Fahad Medical City (Ref #: 22-598E and dated 8 January 2023). Informed consent was obtained from all participants, who were provided with detailed information on the study procedures and various activities conducted during the workshops. Additionally, the participants were assured that their data would be treated with the utmost confidentiality and accessible only to the research team members. Ethical consideration regarding the participants in the control group was addressed by providing an alternative routine care. Additionally, all participants, including those in the control group, were compensated for their participation. Their decision to continue or discontinue their involvement in the study was also respected.

## 3. Results

### 3.1. Enrollment and Demographics

**Figure 1:**
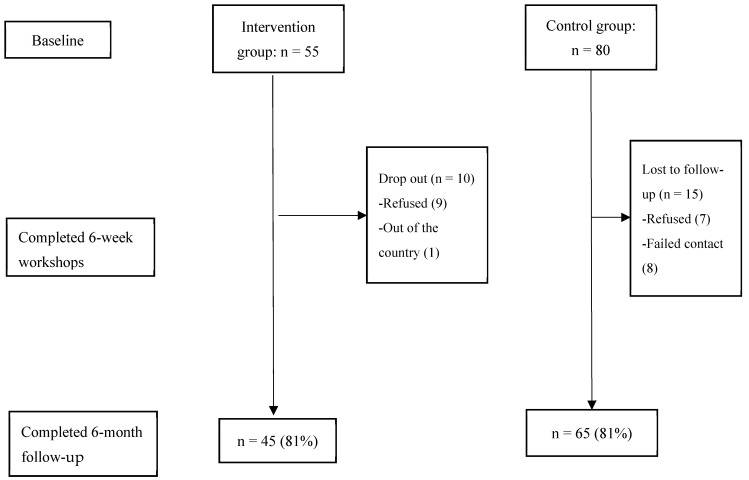
Flow diagram of study participants.

Between October 2022 and April 2023, three CDSMP workshops (with class sizes ranging between 12 and 18) were conducted in Arabic by four trained bilingual leaders at King Saud University. The follow-up of the participants’ recruitment is shown in Figure 1.

Table 1 presents the demographic characteristics of this study’s participants (n = 110). Overall, the response rates for this community-based program study were satisfactory at the baseline and 6-month follow-up, with 78% (n = 135) and 81% (n = 110) response rates, respectively. Overall, the female participants (56.4%) had a mean age of 47.20 years. Of the participants, 67.30% lived with one chronic condition, and 45.5% had less than a bachelor’s education level. The participants (90%) in the intervention group attended ≥4 CDSMP sessions.

### 3.2. Baseline Characteristics

Compared with the control group, the intervention group was significantly younger (43.76 vs. 49.58, *p* < 0.001), had higher education levels (53.3% vs. 46.7%, *p* < 0.01), and fewer participants had low-income levels and fewer medications (20.6% vs. 79.4%, *p* < 0.001; 2.07 vs. 3.34, *p* < 0.01, respectively). No significant differences were found between the groups in other demographic characteristics. No significant differences were found in self-management behaviors (*t* = 0.99, *p* = 0.324) or self-efficacy (*t* = 1.68, *p* = 0.09) between the groups. Additionally, no significant differences between the groups were found at the baseline in any health outcome (cognitive symptom management, illness intrusiveness, pain severity, and depression) (see Table 2).

### 3.3. Comparison between the Groups at 6 Months

After adjusting for covariates, the impact of the intervention was evaluated regarding the following outcomes: (1) self-management behaviors, (2) self-efficacy, and (3) health outcomes 6 months postintervention (Table 3). Using the ANCOVA, the participants in the intervention group had significantly higher scores than did those in the control group for self-management behaviors (F = 11.17, *p* < 0.01) and self-efficacy (F = 9.80, *p* < 0.01). In addition, the intervention group demonstrated better results in all health outcomes. Depression (F = 16.23, *p* < 0.001), and pain severity (F = 8.21, *p* < 0.01) significantly decreased, whereas illness intrusiveness adaptation (F = 12.75, *p* < 0.01) and cognitive symptom management (F = 26.50, *p* < 0.001) significantly increased in the intervention group.

The results of paired *t*-test showed that the experimental group had a statistically significant difference in terms of mean changes in self-management behaviors, self-efficacy, and health outcomes within 6 months after the intervention (see Table 4). Accordingly, the self-management behaviors and self-efficacy scores in the intervention group increased significantly (*p* < 0.001 and *p* < 0.05, respectively), whereas in the control group, they did not significantly change over this 6-month period (*p* = 0.72 and *p* = 0.09, respectively).

## 4. Discussion

This study indicated that the participants in the intervention group achieved significant changes in self-efficacy in managing chronic illnesses, self-management behaviors, and health outcomes (cognitive symptom management, illness intrusiveness, pain severity, and depression). The results of this study suggest that the CDSMP, led by either experts or trained professionals, was effective among Saudi Arabians with chronic diseases, and the impact lasted for ≥6 months. The findings of this study confirmed the results of research conducted in Western, Hispanic, and Asian populations, where implementation of CDSMPs in primary care settings contributes to positive health outcomes and successful chronic disease self-management and control [34,36,39].

The results of the present study showed that the intervention group had statistically significant improvements in terms of the mean scores of self-efficacy, self-management behaviors, and health outcomes 6 months after completing the Stanford CDSMP. In addition, the comparison between the intervention and control groups demonstrated that the effects of the CDSMP were more significant compared with those in the control group who received usual care. For example, the intervention group significantly outperformed the control group on self-efficacy. This improvement may be attributed to the integration of Bandura’s self-efficacy theory into the CDSMP, which had a pivotal influence on the self-efficacy in participants in chronic disease self-management [19]. Patients with high self-efficacy levels are more likely to engage in self-management activities and report health-related improvements [40]. In this study, the participants who attended the CDSMP sessions learned several skills that increased their self-efficacy. These include mechanisms for dealing with stress and negative symptoms, eating habits, making effective decisions, and incorporating physical exercise into daily life. The patients who attended CDSMP workshops were motivated to incorporate these techniques in promoting their confidence levels, leading to successful chronic disease self-management and better health status. In addition, all program sessions underscored the importance of developing weekly action plans and problem-solving skills, enabling the participants to set appropriate goals to improve their health behaviors. Such repetitive activities could have a major impact on self-efficacy, leading to sustainable long-term behavioral changes [40].

Regarding the outcome domain of self-management behaviors, the CDSMP group showed significant increases in adopting self-management activities (physical activities, medication adherence, healthy eating, follow-up visits, and stress management). These findings indicated that the CDSMP was effective in helping participants adopt necessary healthy behaviors. As explained earlier, the participants who underwent the CDSMP learned self-management strategies to develop efficacy in successfully managing chronic conditions. The program primarily focuses on providing patients with the skills required to make life-improving changes, leading to better health outcomes [41]. For example, the participants learned the skills needed to develop plans aimed to achieve specific outcomes and participating in specific self-management behaviors. Their confidence levels increased to mitigate the physical and emotional influences of illness with and without the assistance of healthcare professionals.

Regarding health outcomes, the participants who underwent the CDSMP experienced significant increases in cognitive symptom management. Significant decreases in pain severity, illness intrusiveness, and depression were observed in the CDSMP group. These findings are consistent with those of studies that assessed the impact of community-based programs on health-related outcomes [36,39,40,42]. The patients with chronic conditions, such as diabetes, hypertension, and arthritis, have high pain levels, unpleasant symptoms, and depressive episodes. Effective self-management and coping strategies can help these patients observe significant improvements in pain and physical comfort. For example, the participants who underwent the CDSMP used several cognitive and coping strategies (including mind distraction, relaxation, positive thinking, and deep breathing techniques) to manage illness symptoms, such as physical fatigue and pain, and reduce stress levels. Notably, the CDSMP was effective in helping participants successfully manage their chronic illnesses.

This study has some limitations. First, this study had a quasi-experimental design by which no participant randomization was conducted. Such issues may have led to potential biases influenced by factors such as the patients’ preferences or research team members’ decisions; particularly, they were the ones who implemented the study intervention and data collection. Second, a quasi-experimental study design limits control over confounding factors compared with randomized experimental studies [43]. This may have made isolating the effects of this experiment on the outcomes difficult. Self-reported questionnaire use is another limitation that may have introduced measurement bias. Furthermore, this study was conducted only in Riyadh. Therefore, implementation of this nationwide is recommended for future research. To provide reliable and supportive knowledge, acknowledging these limitations when interpreting the results is important.

Implementation of chronic disease self-management interventions in community settings is not new. Studies have reported the profound benefits of community-based programs to patients with chronic diseases. However, the concept of community-based chronic disease self-management has not been extensively studied in Saudi Arabia. In the primary care settings, the healthcare system in Saudi Arabia should focus on a patient-centered approach rather than preventive coordinated care for individuals with complex healthcare needs. Studies conducted in Saudi Arabia have indicated that patients in primary care were not equipped with the skills necessary to successfully manage their chronic conditions.

The findings of this study indicated that the Stanford CDSMP could provide a novel approach for facilitating behavioral changes and improving chronic disease control. Unlike other community-based interventions, the Stanford Model focuses on patient care and empowers individuals to effectively manage their chronic diseases through interactive sessions. Rather than solely providing medical information, the program emphasizes teaching participants the necessary skills to manage their condition and play an active role in chronic disease management. Person-centered programs such as the Stanford CDSMP can be regularly implemented in primary care centers, allowing individuals with chronic diseases in Saudi Arabia to take control of their health and choose care appropriate to their needs. Additionally, primary healthcare professionals can lead programs and assist patients in acquiring the self-management behaviors required for successfully managing their chronic diseases.

## 5. Conclusions

The findings of this study could confirm that the CDSMP was an effective method for improving chronic disease self-management behaviors, enhancing self-efficacy, and developing health-related outcomes among people with chronic conditions. This program could also effectively empower people to take control of their health conditions, enhance their well-being, and reduce healthcare burdens on hospitals and PHCs. Future research should focus on determining the effectiveness of this program in managing specific diseases and/or identifying opportunities for program implementation in different populations and healthcare contexts.

## Figures and Tables

**Table 1:** Individuals’ characteristics.

	Intervention (n = 45)	Control (n = 65)	x^2^/*t*-Value
Mean Age (SD)	43.40 (6.94)	48.97 (14.58)	2.76 ***
Sex			5.23 *
Female (%)	50	50	
Male (%)	29.2	70.8	
Education			8.43 **
Higher Education (%)	46.3	53.7	
Lower Education (%)	72.3	27.7	
Income			30.43 ***
<9000 Riyals (%)	22.2	77.8	
>9000 Riyals (%)	72.5	27.5	
Mean Medication Numbers (SD)	2 (1.19)	3.35 (2.30)	3.77 **
Mean History of Chronic Diseases in Years (SD)	9.95 (8.60)	9.29 (7.21)	0.40
Comorbidity			
Living with One Chronic Disease (%)	47.2	52.8	2.37
Living with ≥One Chronic Disease (%)	29	71	
Disease Management Support			10.03 **
Family Support (%)	21.1	78.9	
By the Patient (%)	53.8	46.2	

Note: Independent *t*-test or chi-square test analyses were performed to compare characteristics between groups. SD: standard deviation; * *p* < 0.05; ** *p* < 0.01; *** *p* < 0.001.

**Table 2:** Baseline mean scores of outcome measures.

Outcome Measures	Mean (SD)		*t*-Value
	Intervention	Control	
Self-Management Behaviors (range 19–76) ^$^	47.65 (9.73)	50.25 (9.90)	0.99
Self-Efficacy (range 6–60) ^$^	34.72 (13.64)	33.10 (11.75)	1.68
Health-Related Outcomes			
Cognitive Symptom Management (range 0–30) ^$^	13.60 (4.69)	12.26 (6.40)	1.88
Illness Intrusiveness (range 13–91) ^$^	57.25 (16.52)	51.36 (15.67)	1.56
Pain Severity (range 5–38) ^§^	20.04 (6.36)	17.08 (8.24)	1.95
Depressive Symptom (range 0–24) ^§^	9.27 (2.98)	6.41 (5.77)	2.82 **

Note: Independent *t*-test analyses were conducted to compare baseline mean scores between intervention and control groups; The brackets indicate the range of score; ^$^ A higher score is better; ^§^ A lower score is better. ** *p* < 0.01.

**Table 3:** Comparison of outcomes between the intervention (CDSMP) and control (usual care) groups using ANCOVA.

Outcome Measure	Group	Mean	S.E.	95% CI	F-Value
Self-Management Behaviors (range 19–76) ^$^	Intervention	54.38	0.97	52.45–56.30	11.17 **
	Control	49.84	0.82	48.21–51.46	
Self-Efficacy (range 6–60) ^$^	Intervention	40.21	1.29	37.63–42.79	8.80 **
	Control	34.88	1.10	32.69–37.06	
Health-related Outcomes					
Cognitive Symptom Management (range 0–30) ^$^	Intervention	17.22	0.76	15.71–18.74	26.50 ***
	Control	12.27	0.64	10.99–13.55	
Illness Intrusiveness (range 13–91) ^$^	Intervention	61.17	1.80	57.58–64.76	12.76 **
	Control	51.65	1.52	48.63–54.68	
Pain Severity (range 5–38) ^§^	Intervention	14.20	0.88	12.45–15.95	8.22 **
	Control	17.32	0.77	15.84–18.79	
Depressive Symptom (range 0–24) ^§^	Intervention	4.16	0.46	3.23–5.08	16.22 ***
	Control	7.35	0.39	6.57–8.12	

Analysis of covariance on the mean changes of scores at 6 months, controlling for baseline scores of outcome measures; The brackets indicate the range of score; ^$^ A higher score is better; ^§^ A lower score is better; ** *p* < 0.01; *** *p* < 0.001.

**Table 4:** Paired sample *t*-test on outcome measures.

Outcome Measures	Intervention			Control		
	Pretest Mean (SD)	Posttest Mean (SD)	*t*-Test	Pretest Mean (SD)	Posttest Mean (SD)	*t*-Test
Self-Management Behaviors (range 19–76) ^$^	47.65 (9.73)	53.76 (8.03)	3.91 ***	50.25 (9.90)	50.12 (8.85)	0.35
Self-Efficacy (range 6–60) ^$^	34.72 (13.64)	40.04 (12.95)	1.98 *	33.10 (11.75)	33.95 (11.47)	1.71
Cognitive Symptom Management (range 0–30) ^$^	13.60 (4.69)	17.11 (5.60)	4.10 ***	12.26 (6.40)	11.46 (6.52)	0.48
Illness Intrusiveness (range 13–91) ^$^	57.25 (16.52)	61.06 (17.27)	2.28 *	51.36 (15.67)	49.83 (14.51)	0.66
Pain Severity (range 5–38) ^§^	20.04 (6.36)	15.02 (3.46)	4.52 ***	17.08 (8.24)	16.40 (8.57)	0.65
Depressive Symptom (range 0–24) ^§^	9.27 (2.98)	5.06 (3.28)	8.01 ***	6.41 (5.77)	6.53 (4.81)	0.65

Note: Paired *t*-test analyses were performed to compare outcome scores within groups. The brackets indicate the range of score. SD: standard deviation; ^$^ A higher score is better; ^§^ A lower score is better; * *p* < 0.05; *** *p* < 0.001.

## Data Availability

Data are not shared due to privacy and ethical restrictions.
